# Reassessing Joint Use Agreements to Promote the Public’s Health

**DOI:** 10.5888/pcd12.150135

**Published:** 2015-04-16

**Authors:** James G. Hodge

New paradigms in public health practice focus on “health in all policies”(1) or the more encompassing “culture of health”(2) espoused recently by the Robert Wood Johnson Foundation. Under these expansive views of the role of public health in society, interventions that bridge public and private efforts to maximize the use of limited resources make good sense. Years ago, as national obesity rates began to escalate, federal, state, and local public health policy makers sought innovative ways to increase physical activity to counter downward trends at home and work. Encouraging Americans to get active is not so easy when there is a lack of safe places to do so, especially for those living in dense, urban areas sometimes devoid of parks or suitable open spaces. In these and other communities, schools have traditionally been used for community sports and other activities. Yet school grounds and facilities are often gated and locked after hours to protect school property, teachers, administrators, and students. Consequentially, access to these facilities in many jurisdictions can be cut off during the school year or for entire summers when schools are largely out of session. The simple solution in many places is to foster open use arrangements allowing for greater use of school properties for recreation and other healthy activities.

Unfortunately, as Everett Jones and Wendel (3) and Stein et al (4) note in this issue of *Preventing Chronic Disease*, this solution may not be so simple after all. Their research and analyses illustrate how legal, policy, and other barriers can limit the ability of schools and public health actors to secure open use access to indoor and outdoor school grounds. The reasons are manifold. Some schools see little need to make their facilities available for nonschool-related recreation or activities, especially in places where other open-space options exist. Costs related to school property maintenance and upkeep in support of open uses may stretch limited education budgets. Legal concerns over potential liability for injuries or other harms occurring after hours on school grounds raise school administrators’ fears (even though these risks are based more in perception than reality).

These and other concerns can be addressed through joint use agreements (JUAs) that outline specific terms of collaboration between municipal health authorities and schools for the community’s use of schools’ open spaces. JUAs can be effective tools to formally encourage schools to contribute to their community’s health by lending their land and facilities. As Stein et al note, the Centers for Disease Control and Prevention (CDC) incorporated JUAs as a strategic element of its Community Transformation Grants (CTGs) beginning in 2011. National uptake in the use of JUAs, however, has been challenging, as described by CDC researchers Everett Jones and Wendel (3). They compared self-reported survey data from a representative sample of school districts with existing information on economic, race-based, and metropolitan factors related to these districts.

Their findings are revealing. Only 61.6% of sampled school districts had entered into any formal JUAs. Among those executing JUAs, the breadth and terms of the agreements varied extensively on factors such as 1) access to indoor or outdoor space, 2) types of facilities covered (eg, recreation spaces, libraries, health facilities), 3) size and location of the districts, 4) metropolitan classification, and 5) percentage of students receiving free or reduced-price lunches. JUAs are more frequent in urban areas in Western states than in rural areas in Southern states. Although the researchers could not conclude in this study whether executed JUAs have actually led to greater use of school spaces across districts, their conclusions about potential impediments to their use are profound. Some schools lack adequate space to donate for community uses. Others fail to anticipate how a community could use their space when designing buildings or grounds. School districts may not have enough personnel to execute or administer JUAs. Some states’ laws do not support community use of school spaces. According to the authors, in 2010, only 8 states legally required schools to open their spaces for community uses, although 37 states and the District of Columbia purportedly allow it.

These reported barriers are significant, but Stein et al reveal more problems through their case study analyses of JUA hurdles among CTG grantees in North Carolina, Illinois, and Wisconsin. Many of their findings comport with survey data gathered by Everett Jones and Wendel (3). However, Stein et al suggest that among the most compelling factors limiting the usefulness of JUAs are the agreements themselves. To the extent that they formalize via contract what may previously have been generally agreed upon principles between schools and municipalities, JUAs may actually discourage the continued use of open spaces. In North Carolina, initial attempts to require execution of JUAs as a metric of success for CTG recipients garnered resistance. It led CDC to reconsider and allow evidence of open use policies, in addition to formal JUAs, as a metric for grantees.

Other problems with JUAs surfaced according to Stein et al. Prospective or actual parties lacked real-world examples of model JUAs, were concerned about the formality and legality of the deals, or could not understand their terms particularly on sensitive issues like liability. One can hardly blame school officials for their concern over key terms in JUAs. Even a casual review of existing JUAs illustrates how legal jargon may confuse personnel without legal training. Consider the following language on potential liability in a publicly available JUA between the City of Baldwin, California, and the Baldwin Park Unified School District (5):

*District Indemnification of City and Assumption of Risk*. . . . [The] District assumes all liability for any injuries, damages, claims, demands, causes of actions that occur during the hours and days that the District has responsibility for the management and operation of the Facilities under this Agreement including liability created or otherwise related to an alleged dangerous condition of public property. To the maximum extent permitted by law, District agrees to hold harmless, defend, and indemnify City, its city council, agents, officers, employees and representatives against all actions, claims, or demands for injury, death, loss, or damages regardless of fault or cause, by anyone whomsoever (except to the extent that such injury, death, loss, or damage was due to the negligent or willful acts or omissions of City, its city council, agents, officers, employees and representatives), whenever such injury, death, loss, damage, or claim is a consequence of, or arises out of, or is incidental to, the use by the District of the Facilities during the hours and days that the District is responsible for the management and operation of the Facilities. . . .

Even seasoned attorneys might struggle to understand when a school district is actually liable under this language; school administrators stand little chance of digesting the meaning of such provisions. Of course, thousands of JUAs executed across the country may feature clearer wording (6). Yet complex legal language may lead school officials to avoid JUA negotiations altogether and seek alternative, nonlegal strategies, as espoused among some administrators in Illinois.

In Wisconsin, legislative attempts to clarify the extent of liability for schools willing to advance open use arrangements via JUAs backfired when state legislators failed to provide adequate liability protections. Stein et al discuss how Wisconsin’s Open Gym Act requires schools to specify open uses of property for which liability protections would apply. Without such specification, schools may be subject to liability claims. Attempts to revise existing JUAs to clarify these uses were sometimes rebuffed by school administrators, effectively shifting liability to community partners operating or programming activities on schools’ open grounds. Everyone seemed to lose in the balance, although alternative strategies with faith-based and other organizations were pursued.

Characterizing JUAs as “imperfect tools for public health practitioners,” Stein et al label them confusing, complex, and infeasible. Worse yet are the potential collateral consequences of JUA execution when schools walk away altogether from long-standing policies allowing open uses. Communities may fail to consider other options for improving access to open spaces that facilitate physical activities. In combination with the statistical analyses of the limited use of these agreements proffered by Everett Jones and Wendel, the role of JUAs nationally is worth a second look. As a singular legal strategy to open school spaces for community use, JUAs have their flaws. However, they are a core part of state and local efforts to increase opportunities for Americans, especially in lower-income, urban areas, for recreation and other activities. With refined legislative support among state and local officials and improved drafting, JUAs may continue to be one of the easier paths to assuring the public’s health through efficient use of existing community resources.

## References

[1] Hodge JG . Public health law in a nutshell, 4. St Paul (MN): West Academic Publishing; 2014.

[2] Plough AL . Building a culture of health: challenges for the public health workforce. Am J Prev Med 2014;47(5, Suppl 3):S388–90. http://www.ajpmonline.org/article/S0749-3797(14)00404-8/fulltext 10.1016/j.amepre.2014.07.037 25439263

[3] Everett Jones S , Wendel A . Characteristics of joint use agreements in school districts in the United States: findings from the School Health Policies and Practices study 2012. Prev Chronic Dis 2015;12:140560. 10.5888/pcd12.140560 PMC4415430 25880769

[4] Stein A , Baldyga W , Hilgendorf A , Walker J , Hewson D , Rhew L , Challenges in promoting joint use agreements: experiences from community transformation grant awardees in North Carolina, Illinois, and Wisconsin, 2011–2014. Prev Chronic Dis 2015;12:140457. 10.5888/pcd12.140457 PMC4415413 25880770

[5] City of Baldwin, California; Baldwin Park Unified School District. Joint-use agreement for shared use of facilities. 2010. http://www.baldwinpark.com/index.php?option=com_docman&task=doc_download&gid=946. Accessed February 18, 2015.

[6] Seattle School District No. 1; Seattle Parks & Recreation, Washington. An agreement for the joint use of facilities between Seattle School District No. 1 and Seattle Parks and Recreation. 2010. http://www.seattle.gov/parks/publications/policy/jua.pdf. Accessed February 18, 2015.

